# Histone demethylase JMJD2D promotes the self-renewal of liver cancer stem-like cells by enhancing EpCAM and Sox9 expression

**DOI:** 10.1074/jbc.RA120.015335

**Published:** 2020-12-03

**Authors:** Yuan Deng, Ming Li, Minghui Zhuo, Peng Guo, Qiang Chen, Pingli Mo, Wengang Li, Chundong Yu

**Affiliations:** 1 State Key Laboratory of Cellular Stress Biology, Innovation Center for Cell Biology, School of Life Sciences, Xiamen University, Xiamen, China; 2 Department of Hepatobiliary Surgery, Xiang’an Hospital of Xiamen University, School of Medicine, Xiamen University, China

**Keywords:** JMJD2D, Wnt signaling, Notch signaling, H3K9me3, liver cancer stem-like cell, 5-c-8HQ, ChIP, chromatin immunoprecipitation, Co-IP, coimmunoprecipitation, CSCs, cancer stem-like cells, CTCs, circulating tumor cells, FACS, fluorescence-activated cell sorter, FBS, fetal bovine serum, GFP, green fluorescent protein, LCSCs, liver cancer stem-like cells, SCNT, somatic cell nuclear transfer, TGF-β, transforming growth factor-beta, TBE, TCF4 binding elements

## Abstract

Cancer stem-like cells (CSCs) contribute to the high rate of tumor heterogeneity, metastasis, therapeutic resistance, and recurrence. Histone lysine demethylase 4D (KDM4D or JMJD2D) is highly expressed in colon and liver tumors, where it promotes cancer progression; however, the role of JMJD2D in CSCs remains unclear. Here, we show that JMJD2D expression was increased in liver cancer stem-like cells (LCSCs); downregulation of JMJD2D inhibited the self-renewal of LCSCs *in vitro* and *in vivo* and inhibited the lung metastasis of LCSCs by reducing the survival and the early lung seeding of circulating LCSCs. Mechanistically, JMJD2D promoted LCSC self-renewal by enhancing the expression of CSC markers EpCAM and Sox9; JMJD2D reduced H3K9me3 levels on the promoters of EpCAM and Sox9 to enhance their transcription *via* interaction with β-catenin/TCF4 and Notch1 intracellular domain, respectively. Restoration of EpCAM and Sox9 expression in JMJD2D-knockdown liver cancer cells rescued the self-renewal of LCSCs. Pharmacological inhibition of JMJD2D using 5-c-8HQ reduced the self-renewal of LCSCs and liver cancer progression. Collectively, our findings suggest that JMJD2D promotes LCSC self-renewal by enhancing EpCAM and Sox9 expression *via* Wnt/β-catenin and Notch signaling pathways and is a potential therapeutic target for liver cancer.

Liver cancer represents the second most common cancer-related cause of death worldwide with most cases being diagnosed at an advanced stage (1). Although there are significant developments in clinical therapies and research, the 5-year survival rate of liver cancer has not been improved well (2). Patients with liver cancer often encounter tumor progression, recurrence, and distant metastasis. A subset of cells with stem/progenitor cell features called cancer stem-like cells (CSCs) are responsible for the tumor initiation, growth, metastasis, recurrence, and therapeutic resistance (3, 4). Liver cancer stem-like cells (LCSCs) have been shown to be enriched by several CSCs markers, including CD13 (5), CD133 (6), EpCAM (7, 8), Sox9 (9), OV6 (10), CD44 (11), and CD90 (12). Some CSC markers such as EpCAM and Sox9 play essential roles in maintaining the self-renewal of LCSCs. Extensive signaling pathways (such as Wnt/β-catenin, Notch, Hedgehog, transforming growth factor-beta (TGF-β), and IL6/STAT3 pathways) have been reported to be involved in regulating the expression of CSC markers and promoting the self-renewal of CSCs (13, 14, 15, 16).

Recent advances in epigenomics have illuminated that epigenetic regulation contributes to the self-renewal of CSCs (17). JMJD2D, also known as KDM4D, is a histone demethylase that belongs to JMJD2 family, which has five members (JMJD2A−2E) (18). JMJD2D contains a Jumonji C (JmjC) domain that removes methyl groups from lysine 9 on histone 3 (H3K9), lysine 79 on histone 3 (H3K79), and lysine 26 on histone 1.4 (H1.4K26) (19, 20). JMJD2D has been reported to play important roles in androgen target genes activation, DNA damage repair, DNA replication, somatic cell nuclear transfer (SCNT), colitis, and colorectal, liver, and gastrointestinal stromal cancers (21, 22, 23, 24, 25, 26, 27, 28); however, the role of JMJD2D in CSCs remains unclear.

In the present study, we reported that JMJD2D was upregulated in LCSCs and downregulation of JMJD2D markedly inhibited the self-renewal and proliferation of LCSCs *in vitro* and *in vivo*. Mechanistically, JMJD2D promoted the self-renewal of LCSC through enhancement of EpCAM and Sox9 expression Wnt/β-catenin and Notch signaling pathways. The JMJD2D inhibitor 5-c-8HQ could attenuate the self-renewal of LCSCs *in vitro* and *in vivo*. Our findings indicate that JMJD2D may be a potential therapeutic target against LCSCs.

## Results

### Downregulation of JMJD2D reduces the self-renewal of LCSCs *in vitro*

**Figure 1:**
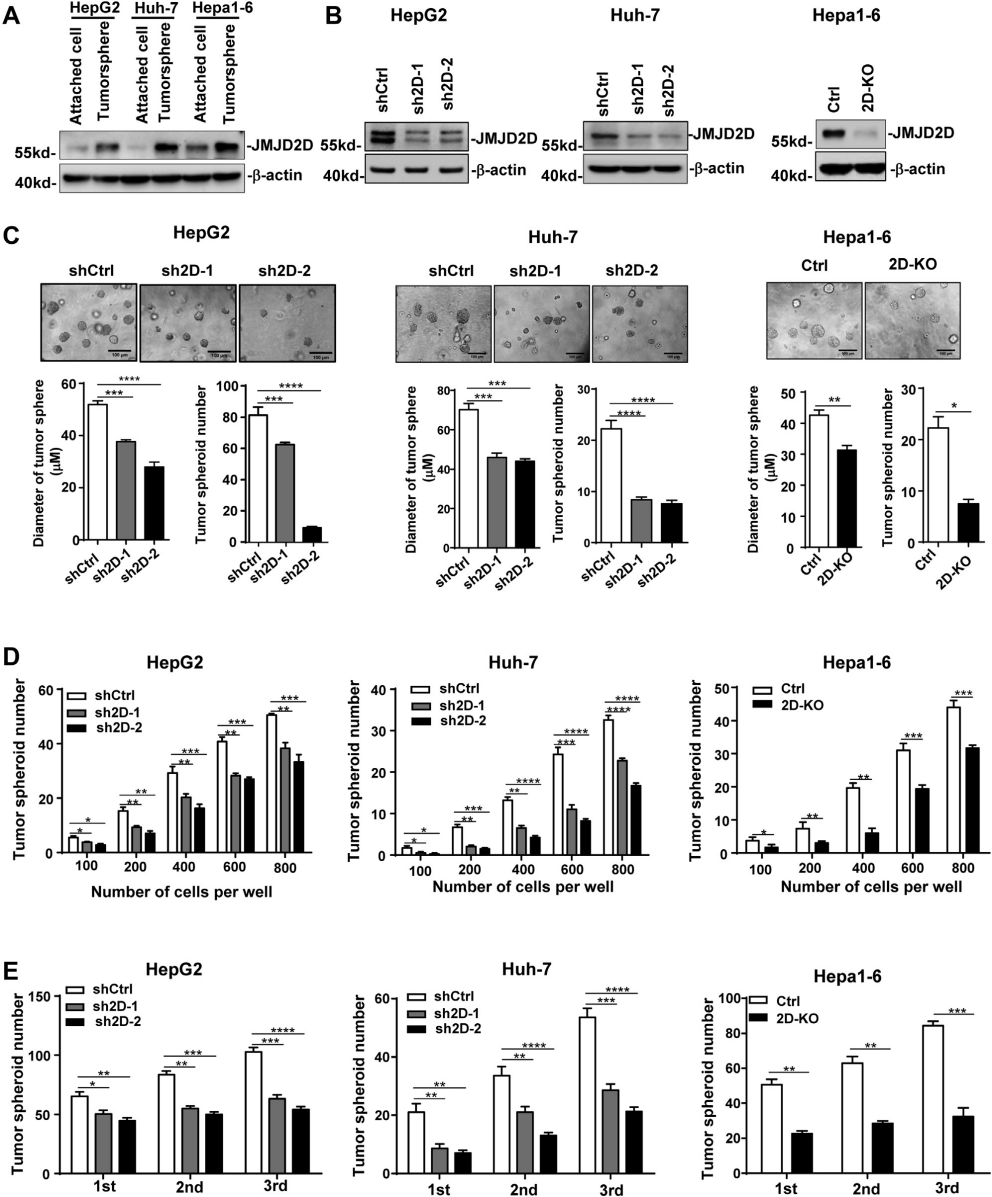
**Downregulation of JMJD2D inhibits the self-renewal of LCSC *in vitro.****A*, JMJD2D protein levels were upregulated in LCSCs compared with non-CSCs (attached cells). *B*, downregulation of JMJD2D protein level by JMJD2D shRNAs and CRISPR-Cas9 system was confirmed by western blot analysis in HepG2, Huh-7, and Hepa1-6. *C*, downregulation of JMJD2D in liver cancer cells reduced tumorsphere number and size. *D*, the *in vitro* limiting dilution assay showed that downregulation of JMJD2D decreased CSC self-renewal frequency. *E*, The serial sphere formation assay showed that downregulation of JMJD2D decreased self-renewal capacities of LCSCs. These experiments were performed at least three times with similar results. ∗, *p* < 0.05; ∗∗, *p* < 0.01; ∗∗∗, *p* < 0.001; ∗∗∗∗, *p* < 0.0001.

We previously reported that JMJD2D was overexpressed in colorectal and liver cancers and promoted cancer progression (25, 26, 27); however，the role of JMJD2D in CSCs remains unclear. Formation of tumorsphere in CSC culture media represents the principal characteristic of CSC self-renewal. To investigate the role of JMJD2D in liver LCSCs, we enriched the LCSCs by inducing hepatoma spheroid formation from human liver cancer cell lines HepG2 and Huh-7 as well as mouse liver cancer cell line Hepa1-6 and then measured the protein and mRNA expression of JMJD2D. As shown in Fig. 1*A* and Fig. S1*A*, both the protein and mRNA levels of JMJD2D were upregulated in LCSCs (tumorsphere) compared with non-CSCs (attached cells), suggesting that JMJD2D may promote the self-renewal of LCSCs. To test this hypothesis, we knocked down JMJD2D in HepG2 and Huh-7 using two different JMJD2D shRNAs (sh2D-1 and sh2D-2) and knocked out JMJD2D in Hepa1-6 using CRISPR-Cas9 system (2D-KO) (Fig. 1*B*) and then performed MTT assay to measure the cell proliferation and tumorsphere formation assay to measure the tumorsphere formation ability. Downregulation of JMJD2D significantly inhibited liver cancer cell proliferation (Fig. S1, *B–D*) and tumorsphere formation ability as demonstrated by reduced tumorsphere number and size (Fig. 1*C*), indicating that JMJD2D can promote the proliferation and tumorsphere formation of liver cancer cells. Furthermore, we employed the limiting dilution assay to determine the CSC self-renewal frequency. At 200, 400, 600, and 800 cell levels, downregulation of JMJD2D resulted in a significant decrease in tumorsphere number (Fig. 1*D*). Serial sphere formation assays validated that the self-renewal capacities of JMJD2D-downregulated cells were decreased as compared with control cells (Fig. 1*E*). Collectively, these results suggest that downregulation of JMJD2D reduces the self-renewal of LCSCs *in vitro*.

### Downregulation of JMJD2D inhibits LCSC-derived tumor initiation and progression *in vivo*

**Figure 2:**
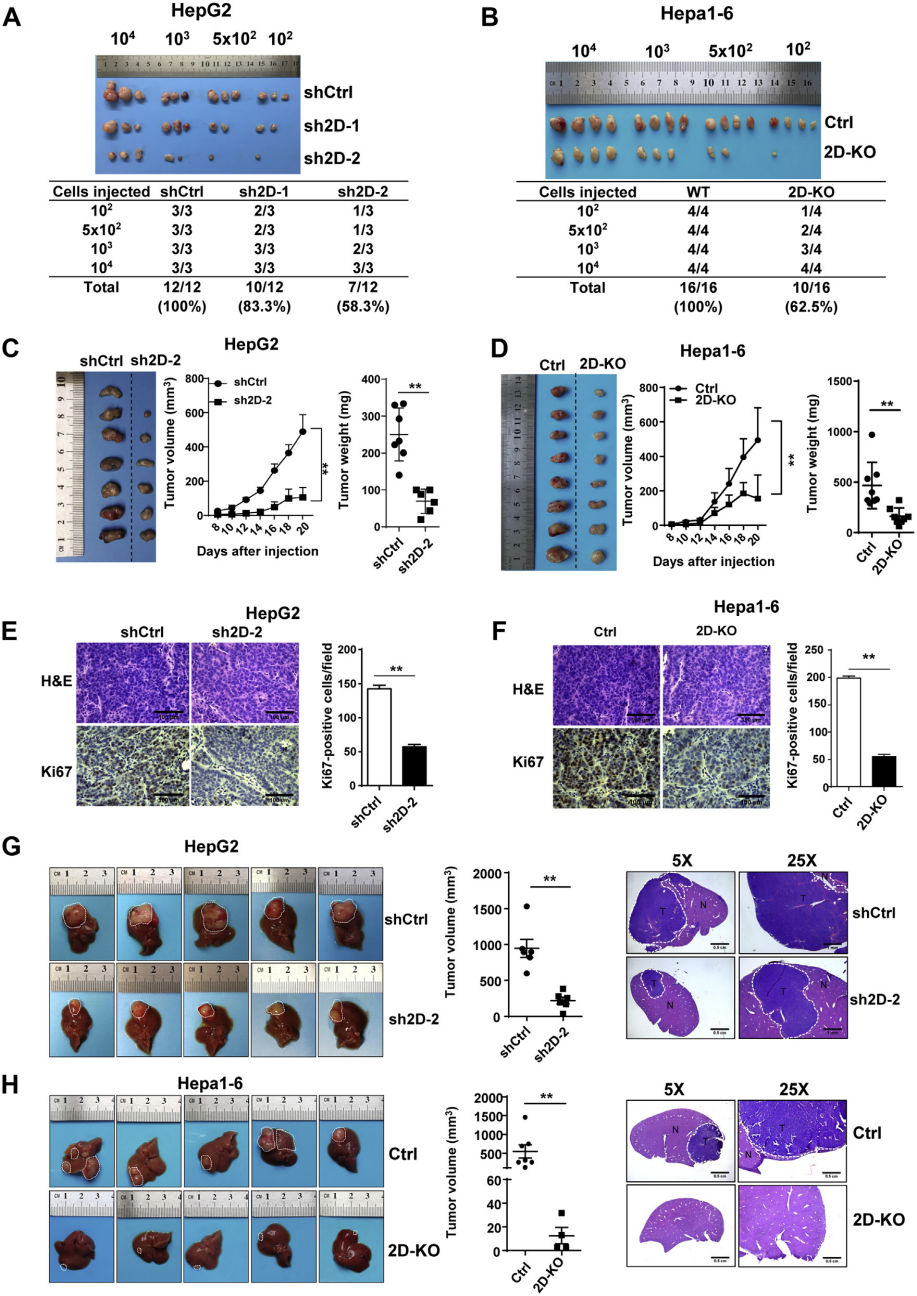
**Dowregulation of JMJD2D inhibits LCSC-derived tumor initiation and progression *in vivo*.***A*–*B*, tumorigenicity of LCSCs was analyzed by subcutaneous injection of LCSCs in nude or C57BL/6 mice (n = 3 or 4/group) and tumor incidence was evaluated after 2 months of implantation (*upper panel*). CSC frequency was calculated by extreme limiting dilution analysis (*lower panel*). *C*–*D*, downregulation of JMJD2D inhibited LCSC-derived subcutaneous graft tumors growth. *E*–*F*, downregulation of JMJD2D inhibited LCSC-derived tumor cell proliferation. H&E staining was performed on the sections of subcutaneous graft tumors. The expression of Ki-67 was determined by IHC on the sections of subcutaneous graft tumors. *G*–*H*, downregulation of JMJD2D inhibited the growth of LCSCs-derived orthotopic graft tumors. N, Normal; T, Tumor. These experiments were performed twice with similar results. ∗∗, *p* < 0.01.

CSCs have a strong ability to form tumors (29). To characterize the role of JMJD2D in LCSC-derived tumor initiation and progression, *in vivo* limiting dilution assay was performed using LCSCs disassociated from cultured spheroids. LCSCs derived from JMJD2D-downregulated spheroids displayed a lower tumorigenicity compared with the cells from control spheroids (Fig. 2, *A*–*B*). LCSCs from JMJD2D-downregulated spheroids exhibited decreased subcutaneous graft tumor growth and tumor weight (Fig. 2, *C*–*D*). Furthermore, we performed Ki67 staining to determine the effects of JMJD2D downregulation on cell proliferation *in vivo*. As shown in Fig. 2, *E*–*F*, downregulation of JMJD2D dramatically reduced Ki67-positive cell number in subcutaneous graft tumors. Although subcutaneous graft tumor is the most common graft tumor model, orthotopic graft tumor model is representative of natural progression of liver cancer. Therefore, we performed orthotopic graft tumor model to determine the effect of JMJD2D downregulation on the initiation and progression of LCSC-derived tumors *in vivo*. We found that orthotopic graft tumors derived from JMJD2D-downregulated spheroids grew much slower than control tumors (Fig. 2, *G*–*H*). These results indicate that downregulation of JMJD2D inhibits the initiation and progression of LCSC-derived tumor *in vivo*.

### Downregulation of JMJD2D inhibits the lung metastasis of LCSCs by reducing the survival and the early lung seeding of circulating LCSCs

**Figure 3:**
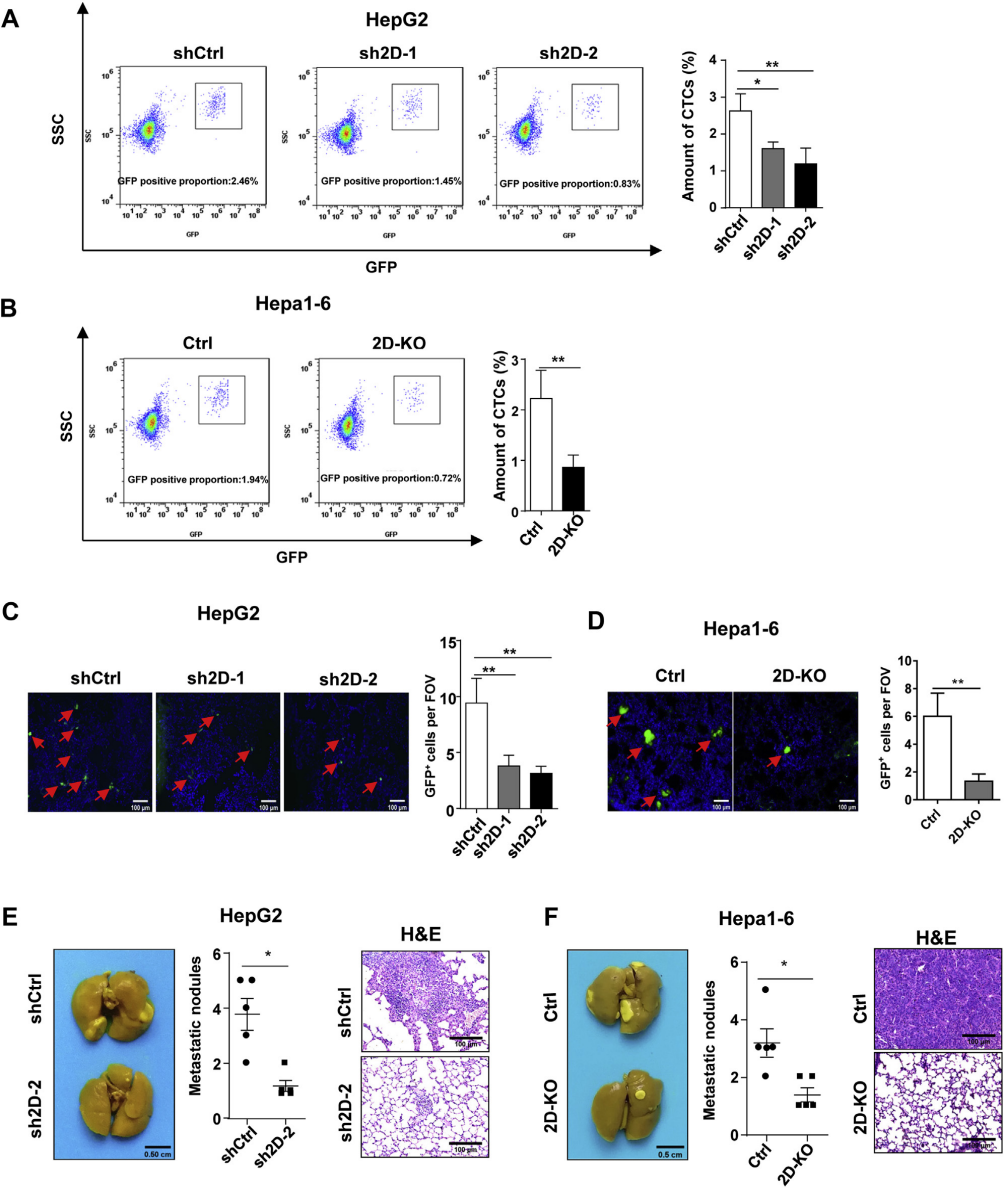
**Downregulation of JMJD2D reduces the survival and the early lung seeding of circulating LCSCs to inhibit the lung metastasis of LCSCs.***A*–*B*, downregulation of JMJD2D inhibits the survival of circulating tumor cells in peripheral blood. The amount of CTCs was determined by detecting GFP using flow cytometry after 36 h of inoculation (n = 5). *C*–*D*, JMJD2D knockdown decreased the number of the early seeding of LCSCs. GFP signal indicated LCSCs in lung. *E* and *F*, downregulation of JMJD2D inhibited the lung metastasis of LCSCs. These experiments were performed twice with similar results. ∗, *p* < 0.05; ∗∗, *p* < 0.01.

Tumor metastasis is the major cause of cancer-associated mortality. Tumor cells invade the surrounding tissue of the primary tumor, intravasate into blood to become circulating tumor cells (CTCs), translocate to distant tissues, and eventually seed, proliferate, and colonize to form metastatic tumors (30). CSCs are regarded to be the source of CTCs and responsible for tumor metastasis. To evaluate the function of JMJD2D in CTCs, we injected green fluorescent protein (GFP)-labeled LCSCs into mouse tail vein to establish a mouse model of CTCs and then detected GFP-labeled CTCs in mouse peripheral blood by flow cytometry as well as counted the early lung seeding GFP-labeled CTCs under the fluorescence microscope at 36 h after LCSCs injection. Downregulation of JMJD2D significantly reduced the number of CTCs in mouse peripheral blood (Fig. 3, *A*–*B*) and the number of the early lung seeding CTCs (Fig. 3, *C*–*D*), suggesting that JMJD2D promotes the survival of CTCs in blood and the early seeding of CTCs in the lung. Consequently, the number of metastasizing tumors in the lungs was significantly decreased in mice injected with JMJD2D-downregulated LCSCs as compared with control cells (Fig. 3, *E* and *F*). These results suggest that downregulation of JMJD2D inhibits the lung metastasis of LCSCs by reducing the survival and the early lung seeding of circulating LCSCs.

### JMJD2D demethylates the H3K9me3 on the promoters of EpCAM and Sox9 to facilitate the recruitment and transactivation of β-catenin/TCF4 and NICD1, respectively

To investigate the molecular mechanisms by which JMJD2D promotes the self-renewal of LCSCs, we examined the effects of JMJD2D knockdown on the expression of several LCSCs-related CSC markers, including CD13, CD44, CD133, OCT4, Sox2, KLF4, Nanog, EpCAM, CD90, and Sox9. As shown in Fig. S2*A*, the mRNA levels of EpCAM and Sox9, but not other CSC markers, were significantly reduced in two JMJD2D-knockdown HepG2 cell lines. Knockdown of either EpCAM or Sox9 significantly inhibited the proliferation and tumorsphere formation ability of HepG2 cells (Fig. S2, *B*–*C*), suggesting that downregulation of EpCAM and Sox9 may, at least in part, be responsible for the inhibitory effects of JMJD2D knockdown on the self-renewal of LCSCs.

**Figure 4:**
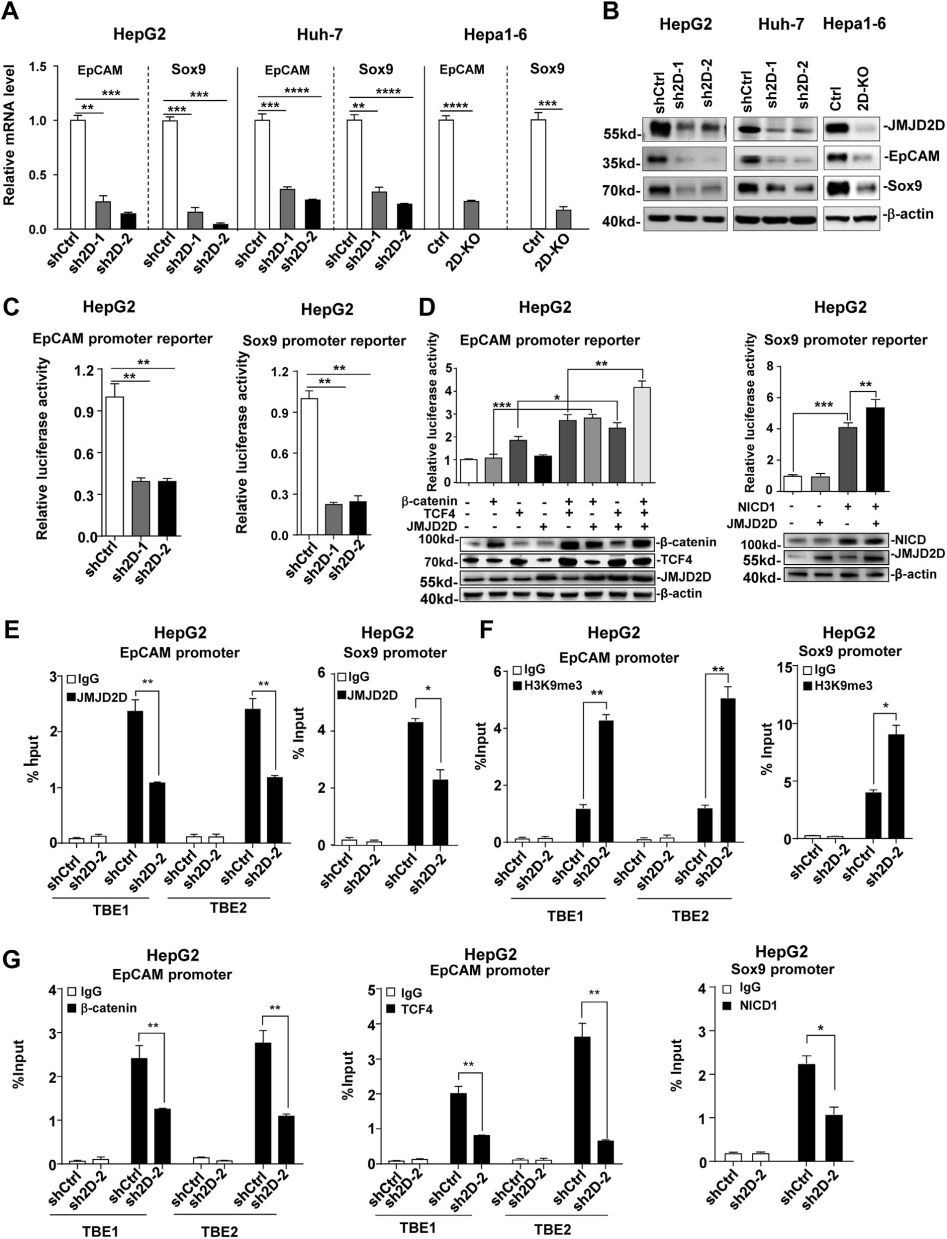
**JMJD2D demethylates the H3K9me3 on the promoters of EpCAM and Sox9 to facilitate the recruitments and transactivations of β-catenin/TCF4 and NICD1, respectively.***A*–*B*, downregulation of JMJD2D reduced the mRNA and protein levels of EpCAM and Sox9 in HepG2, Huh-7, and Hepa1-6. *C*, knockdown of JMJD2D suppressed the promoter activities of EpCAM and Sox9 in HepG2 cells. *D*, JMJD2D cooperated with TCF4/β-catenin or NICD1 to enhance the promoter activities of EpCAM and Sox9 in HepG2 cells, respectively. *E*, knockdown of JMJD2D reduced JMJD2D recruitment to EpCAM and Sox9 promoters. *F*, knockdown of JMJD2D increased the H3K9me3 levels at the EpCAM and Sox9 promoters. *G*, knockdown of JMJD2D reduced β-catenin/TCF4 and NICD1 recruitment to the EpCAM and Sox9 promoters, respectively. TBE: TCF4 binding element. These experiments were performed at least three times with similar results. ∗, *p* < 0.05; ∗∗, *p* < 0.01; ∗∗∗, *p* < 0.001; ∗∗∗∗, *p* < 0.0001.

To show that the inhibitory effects of JMJD2D knockdown on EpCAM and Sox9 expression is not just limited to HepG2 cells, we also measured the mRNA levels of EpCAM and Sox9 in JMJD2D-downregulated Huh-7 and Hepa1-6 cells. As shown in Fig. 4*A*, downregulation of JMJD2D significantly decreased EpCAM and Sox9 mRNA levels in HepG2, Huh-7, and Hepa1-6 cells. Consistent with the mRNA levels, the protein levels of EpCAM and Sox9 were decreased in JMJD2D-downregulated HepG2, Huh7, and Hepa1-6 cells compared with control cells (Fig. 4*B*). Furthermore, we found that the expression of JMJD2D, EpCAM, and Sox9 was upregulated in human liver cancer specimens in GEO database (Fig. S3*A*), and the mRNA levels of JMJD2D were positively correlated with EpCAM and Sox9 in TCGA database (Fig. S3*B*).

To determine whether JMJD2D regulates EpCAM and Sox9 expression at the transcriptional level, we transfected EpCAM and Sox9 promoter reporters into JMJD2D-knockdown and control cells, respectively. The results showed that knockdown of JMJD2D decreased the promoter activities of EpCAM and Sox9 (Fig. 4*C*), suggesting that JMJD2D regulates EpCAM and Sox9 expression at the transcriptional level. It has been reported that EpCAM is a transcriptional target gene of Wnt/β-catenin signaling pathway with two TCF4 binding elements (TBE) on the EpCAM promoter (TBE1 and TBE2) (31), and Sox9 is a transcriptional target gene of Notch signaling pathway with a NICD1 binding site on the Sox9 promoter (32). Therefore, we performed promoter reporter assays to determine whether JMJD2D can cooperate with β-catenin/TCF4 and NICD1 to enhance the promoter activities of EpCAM and Sox9, respectively. As shown in Fig. 4*D*, ectopic expression of JMJD2D and β-catenin/TCF4 as well as JMJD2D and NICD1 synergistically increased the promoter activities of EpCAM and Sox9, respectively, indicating that JMJD2D cooperates with β-catenin/TCF4 and NICD1 to enhance the transcription of EpCAM and Sox9, respectively.

Next, we wondered whether JMJD2D and β-catenin/TCF4 can be recruited to the endogenous EpCAM promoter, and JMJD2D and NICD1 can be recruited to the endogenous Sox9 promoter. To this end, chromatin immunoprecipitation (ChIP) assays were conducted. As shown in Fig. 4*E*, JMJD2D could be recruited to the promoters of EpCAM and Sox9, but JMJD2D knockdown reduced its recruitment as expected. As a histone demethylase, JMJD2D promotes gene transcription through demethylating H3K9me3 on the promoter. The results of ChIP assays showed that the H3K9me3 levels on the promoters of EpCAM and Sox9 were increased in JMJD2D-knockdown cells compared with control cells (Fig. 4*F*), suggesting that JMJD2D is responsible for demethylating H3K9me3 on the promoters of EpCAM and Sox9. The results of ChIP assays showed that β-catenin/TCF4 could be recruited to the EpCAM promoter and NICD1 could be recruited to the Sox9 promoter as expected, but their recruitment was reduced in JMJD2D-knockdown cells (Fig. 4*G*), indicating that H3K9me3 demethylation facilitates the recruitment of β-catenin/TCF4 and NICD1 to the promoters of EpCAM and Sox9, respectively. Collectively, these results suggest that JMJD2D demethylates H3K9me3 on the promoters of EpCAM and Sox9 and facilitates the recruitment and transactivation of TCF4/β-catenin and NICD1, respectively.

To determine whether the demethylase activity of JMJD2D is required for the transcription of EpCAM and Sox9 to promote the self-renewal of LCSCs, we transfected wild-type JMJD2D and demethylase-defective JMJD2D^S200M^ mutant plasmids into HepG2 cells, respectively. Compared with wild-type JMJD2D, JMJD2D^S200M^ mutant failed to cooperate with β-catenin/TCF4 and NICD1 to enhance the promoter activities of EpCAM and Sox9 (Fig. S4*A*), failed to induce the expression of EpCAM and Sox9 in HepG2 cells (Fig. S4*B*), and failed to promote tumorsphere formation of LCSCs (Fig. S4*C*). These results suggest that the demethylase activity is required for JMJD2D to enhance EpCAM and Sox9 expression to promote the self-renewal of LCSCs.

### JMJD2D interacts with β-catenin/TCF4 and NICD1

**Figure 5:**
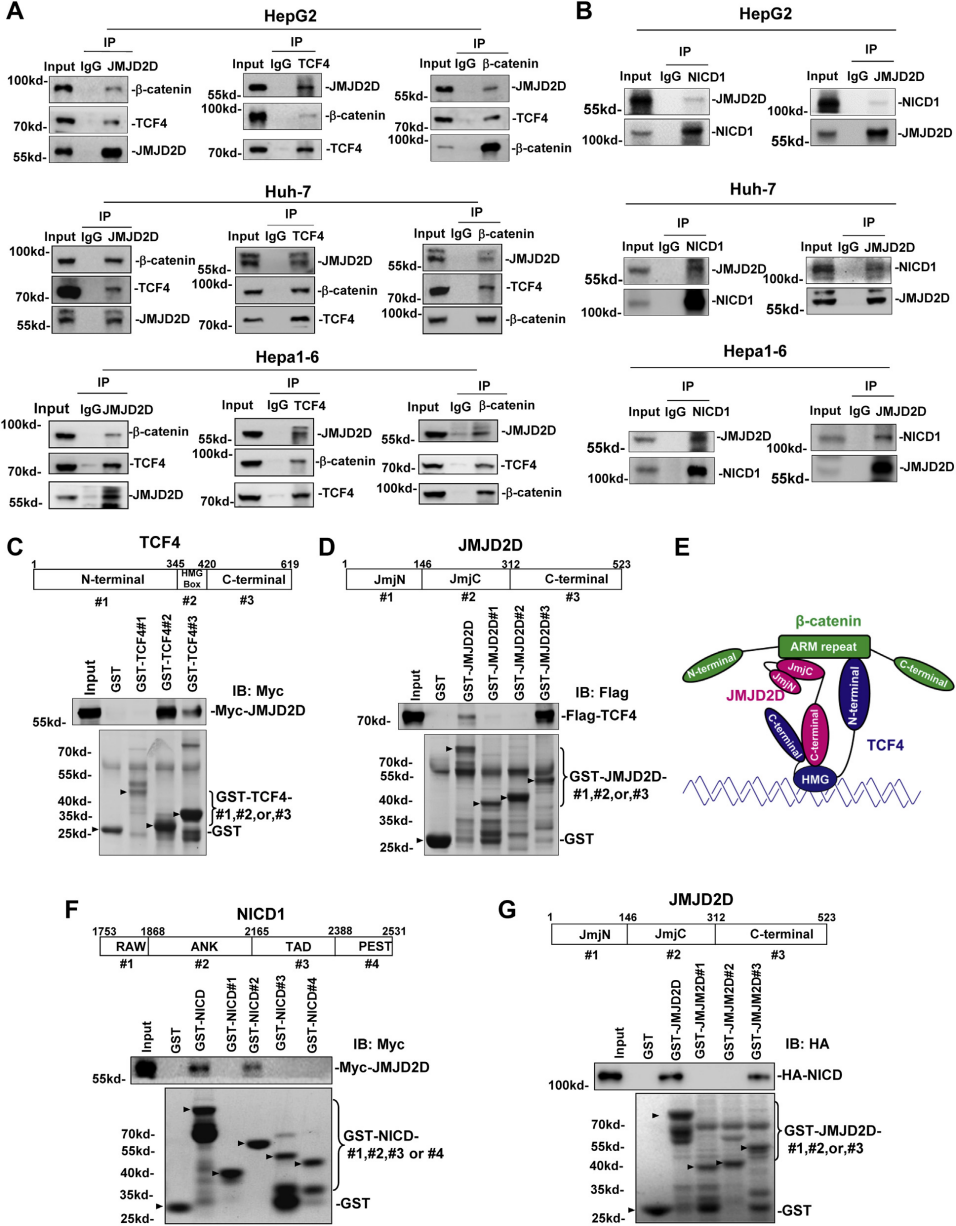
**JMJD2D interacts with β-catenin/TCF4 and NICD1.***A*, JMJD2D interacted with β-catenin and TCF4 in HepG2, Huh-7, and Hepa1-6 cells. The interaction between JMJD2D, β-catenin, and TCF4 was detected by Co-IP assay. *B*, JMJD2D interacted with NICD1 in HepG2, Huh-7, and Hepa1-6 cells. The interaction between JMJD2D and NICD1 was detected by Co-IP assay. *C*, GST pull-down analysis of the interaction between JMJD2D and the different domains of TCF4. *D*, GST pull-down analysis of the interaction between TCF4 and the different domains of JMJD2D. *E*, schematic model of the interaction between JMJD2D, TCF4, and β-catenin. *F*, GST pull-down analysis of the interaction between JMJD2D and the different domains of NICD1. *G*, GST pull-down analysis of the interaction between NICD1 and the different domains of JMJD2D. These experiments were performed at least three times with similar results.

To determine whether JMJD2D could interact with β-catenin/TCF4 and NICD1 in liver cancer cells, we performed Co-IP assays. The results showed that JMJD2D interacted with β-catenin/TCF4 and NICD1 in HepG2, Huh-7, and Hepa1-6, respectively (Fig. 5, *A* and *B*). Furthermore, GST pull-down assays showed that JMJD2D interacted with TCF4 at its HMG Box domain and C-terminal domain (Fig. 5*C*), and TCF4 interacted with JMJD2D at its C-terminal domain (Fig. 5*D*). We previously reported that the Jmjc domain of JMJD2D interacted with the ARM-3-10 domain of β-catenin (33). Thus, JMJD2D bound to β-catenin and TCF4 *via* different domains. The N-terminal domain of TCF4 could interact with the ARM-3-10 domain of β-catenin (Fig. S5, *A* and *B*). Thus, JMJD2D, β-catenin, and TCF4 interact with each other to form a ternary complex (Fig. 5*E*). GST pull-down assays also showed that JMJD2D interacted with NICD1 at its ANK domain (Fig. 5*F*), and NICD1 interacted with JMJD2D at its C-terminal domain (Fig. 5*G*). Collectively, these results implicate that β-catenin/TCF4 could interact with JMJD2D to recruit it to the EpCAM promoter and NICD1 could interact with JMJD2D to recruit it to the Sox9 promoter to enhance EpCAM and Sox9 expression, respectively.

### Ectopic expression of EpCAM and Sox9 rescues the self-renewal of JMJD2D-knockdown LCSCs

**Figure 6:**
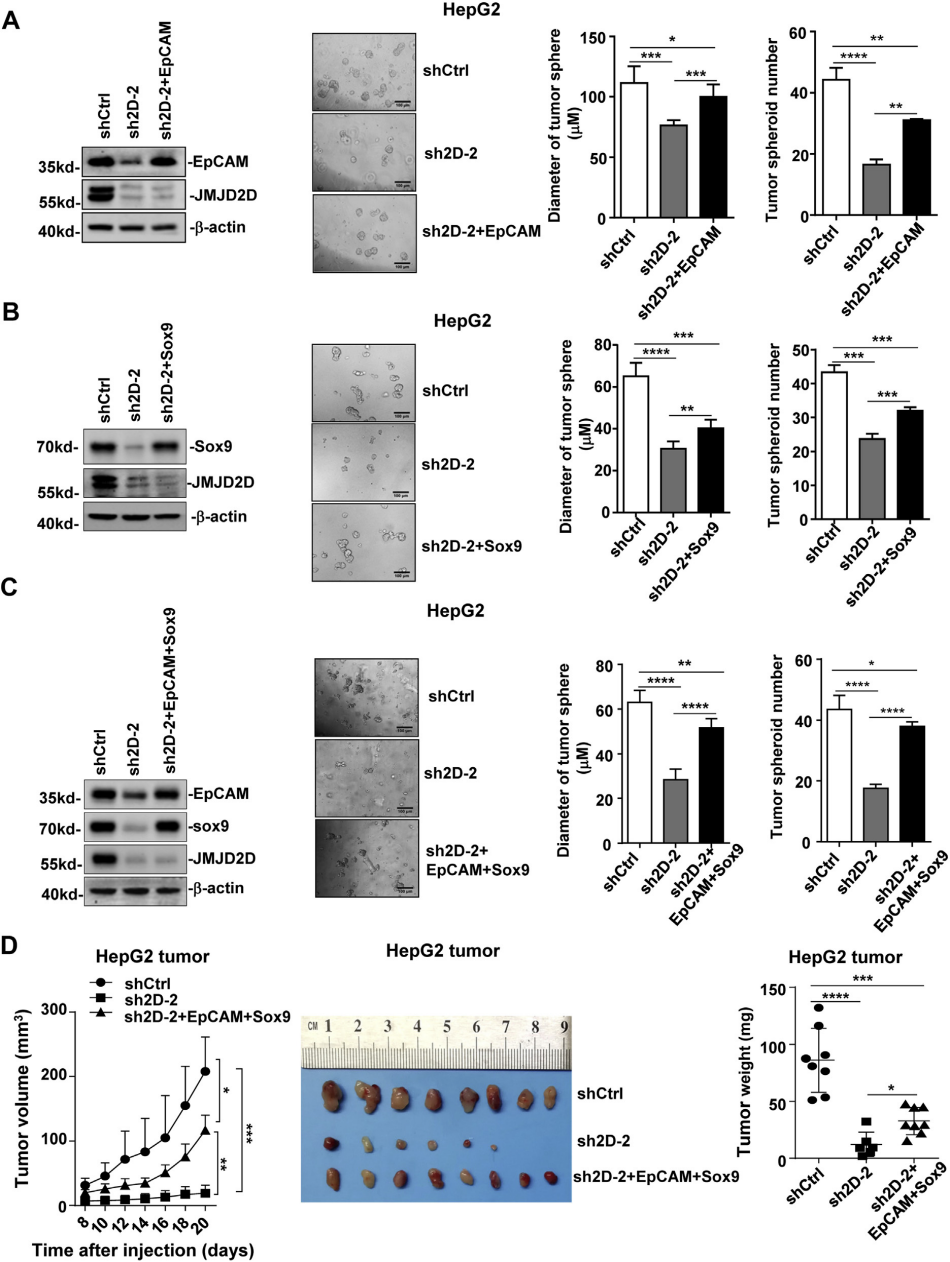
**Ectopic expression of EpCAM and Sox9 rescues the self-renewal ability of JMJD2D-knockdown LCSCs.***A*, Ectopic expression of EpCAM partly rescued the tumorsphere formation ability of JMJD2D-knockdown cells. *B*, Ectopic expression of Sox9 partly rescued the tumorsphere formation ability of JMJD2D-knockdown cells. *C*, ectopic expression of both EpCAM and Sox9 significantly rescued the tumorsphere formation ability of JMJD2D-knockdown cells. *D*, ectopic expression of both EpCAM and Sox9 in JMJD2D-knockdown cells partly rescued subcutaneous tumor growth and tumor weight *in vivo*. These experiments were performed at least twice with similar results. ∗, *p* < 0.05; ∗∗, *p* < 0.01; ∗∗∗, *p* < 0.001; ∗∗∗∗, *p* < 0.0001.

To further confirm that EpCAM and Sox9 mediate the promoting effect of JMJD2D on the self-renewal of LCSCs, we ectopically expressed EpCAM, Sox9, and EpCAM plus Sox9 in JMJD2D-knockdown HepG2 cells, respectively, and then measured the proliferation and tumorsphere formation abilities of cells. Restoration of EpCAM or Sox9 expression alone partially rescued the proliferation (Fig. S6, *A* and *B*) and the tumorsphere formation abilities of JMJD2D-knockdown cells (Fig. 6, *A* and *B*) Restoration of both EpCAM and Sox9 expression more efficiently rescued the proliferation (Fig. S6*C*) and the tumorsphere formation abilities of JMJD2D-knockdown cells (Fig. 6*C*). Consistently, restoration of EpCAM and Sox9 expression in JMJD2D-knockdown LCSCs partially rescued the subcutaneous tumor growth (Fig. 6*D*). Taken together, these results support the notion that JMJD2D promotes the self-renewal of LCSCs through enhancing the expression of EpCAM and Sox9. Since JMJD2D can activate Wnt and Notch signaling pathways, apart from EpCAM and Sox9, JMJD2D may promote LCSC self-renewal by regulating the expression of other CSC-related genes such as c-Myc and Hes1 *via* Wnt and Notch signaling pathways, respectively (Fig. S7).

### Pharmacological inhibition of JMJD2D reduces EpCAM and Sox9 expression and inhibits the self-renewal and tumorigenesis of LCSCs

**Figure 7:**
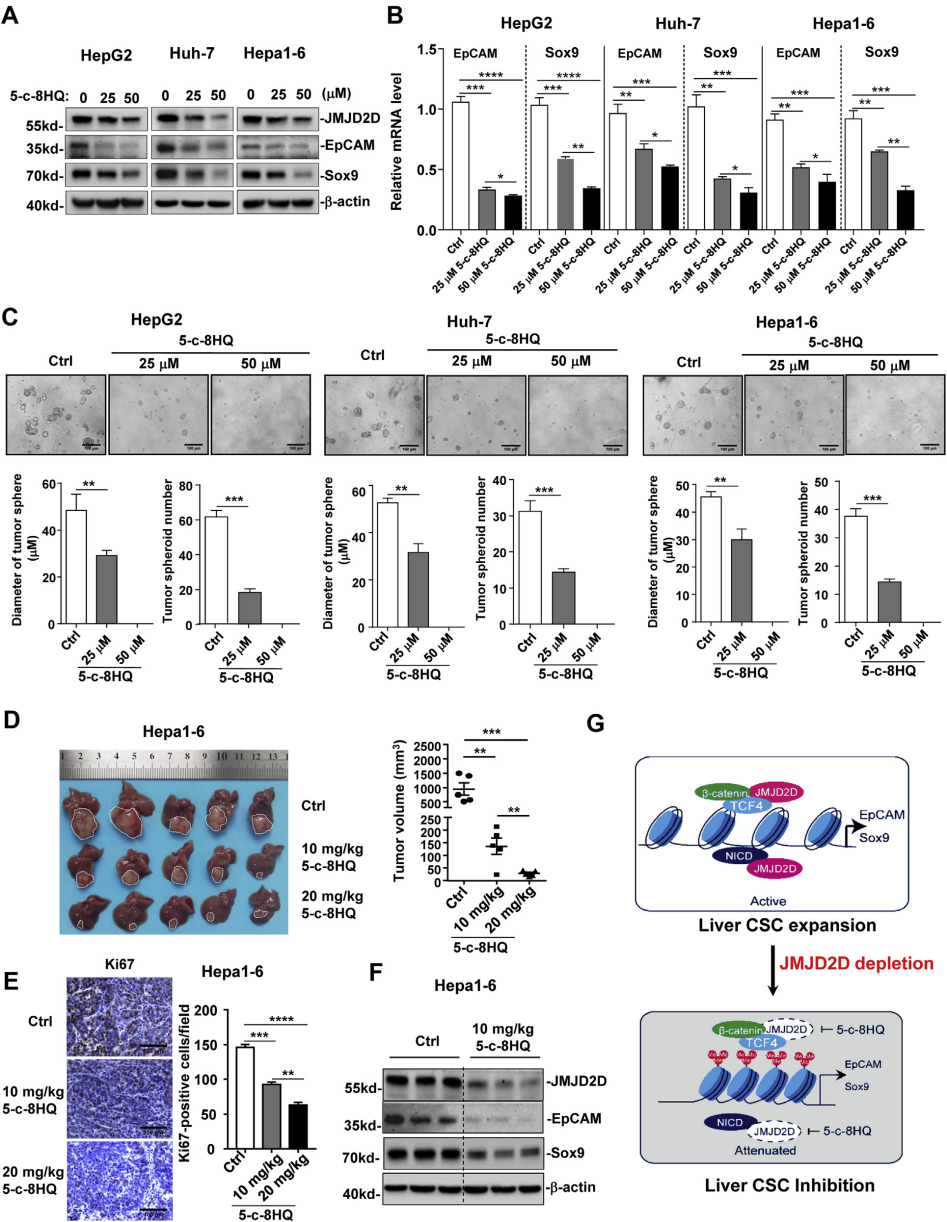
**Pharmacological inhibition of JMJD2D reduces EpCAM and Sox9 expression and inhibits the self-renewal and tumorigenesis of LCSCs.***A*, the JMJD2D inhibitor 5-c-8HQ treatment (48 h) reduced the protein expression levels of JMJD2D, EpCAM, and Sox9 in HepG2, Huh-7, and Hepa1-6 in a dose-dependent manner. *B*, 5-c-8HQ treatment (48 h) reduced the mRNA levels of EpCAM, and Sox9 in HepG2, Huh-7, and Hepa1-6 in a dose-dependent manner. *C*, 5-c-8HQ treatment inhibited the tumorsphere formation abilities of HepG2, Huh-7, and Hepa1-6 in a dose-dependent manner. *D*, 5-c-8HQ (10 mg/kg or 20 mg/kg) treatment inhibited Hepa1-6 orthotopic graft tumor growth *in vivo*. *E*, 5-c-8HQ treatment reduced Ki67 expression in orthotopic graft tumors by IHC assay. After 12 days of 5-c-8HQ administration, mice ware sacrificed, and then the tumors tissues were analyzed by IHC. *F*, 5-c-8HQ treatment reduced JMJD2D, EpCAM, and Sox9 protein expression in orthotopic graft tumors. JMJD2D, EpCAM, and Sox9 protein levels were determined by western blot. *G*, schematic presentation of the mechanisms by which JMJD2D promotes the self-renewal of LCSCs through enhancing EpCAM and Sox9 expression and JMJD2D depletion inhibits EpCAM and Sox9 expression and the self-renewal of LCSCs. These experiments were performed at least twice with similar results.∗, *p* < 0.05; ∗∗, *p* < 0.01; ∗∗∗, *p* < 0.001; ∗∗∗∗, *p* < 0.0001.

Given that downregulation of JMJD2D could inhibit LCSC self-renewal, we wondered whether a JMJD2D inhibitor 5-chloro-8-hydroxyquinoline (5-c-8HQ), which reduces both demythelase activity and protein levels of JMJD2D (26), could inhibit the self-renewal of LCSCs. To this end, HepG2, Huh-7, and Hepa1-6 cells were treated with 5-c-8HQ (Fig. S8*A*), and then the self-renewal of LCSCs was measured by tumorsphere formation assay. As shown in Fig. 7, *A* and *B*, 5-c-8HQ treatment for 48 h significantly reduced the protein and mRNA levels of EpCAM and Sox9 in a dose-dependent manner. Consistently, 5-c-8HQ treatment markedly reduced the tumorsphere formation abilities of LCSCs in a dose-dependent manner (Fig. 7*C*). Furthermore, 5-c-8HQ treatment significantly inhibited LCSC orthotopic graft tumor growth (Fig. 7*D*) and decreased Ki67-positive tumor cells (Fig. 7*E*). As expected, 5-c-8HQ treatment significantly reduced the protein levels of EpCAM and Sox9 in LCSC orthotopic graft tumors (Fig. 7*F*). Moreover, JMJD2D inhibitor 5-c-8HQ could also suppress the expression of c-Myc and Hes1 in orthotopic tumors (Fig. S8*B*). Treatment with 10 mg/kg or 20 mg/kg 5-c-8HQ did not result in obvious adverse effects on mice as demonstrated by no body weight reduction and no toxicity to the major organs after treatment (Fig. S8, *C* and *D*). Collectively, these results demonstrate that JMJD2D could be targeted by a chemical inhibitor to reduce the expression of EpCAM, Sox9, c-Myc, and Hes1 and to inhibit the self-renewal and tumorigenesis of LCSCs (Fig. 7*G*). In addition, transient knockdown of JMJD2D by siRNA reduced the mRNA and protein levels of EpCAM, Sox9, c-Myc, and Hes1 (Fig. S9, *A* and *B*), as well as the proliferation and tumorsphere formation of Hepa1-6 cells (Fig. S9, *C* and *D*), supporting the notion that inhibition of JMJD2D is an effective way to inhibit the self-renewal of LCSCs for liver cancer therapy.

## Discussion

CSCs are believed to be responsible for the initiation, propagation, metastasis, chemoresistance, and heterogeneity of solid tumor (3). Nowadays, increasing evidence shows that epigenetic alterations including DNA methylation and histone modifications participate in CSC self-renewal maintenance (34). Histone-modifying enzymes, such as EZH2, SUV39H1, and LSD1, have been reported to regulate CSC self-renewal (35, 36). In the present study, we demonstrate that histone demethylase JMJD2D promotes LCSC self-renewal and could be a potential therapeutic target against LCSCs as follows: (1) JMJD2D is upregulated in LCSCs; (2) downregulation of JMJD2D inhibits the self-renewal of LCSCs *in vitro* and *in vivo*; (3) pharmacological inhibition of JMJD2D reduces LCSC self-renewal and tumorigenesis.

EpCAM and Sox9 could enhance the self-renewal, propagation, and metastasis ability of LCSCs (8, 9). In this study, we found that downregulation of JMJD2D reduced the expression of LCSC biomarkers EpCAM and Sox9 and inhibited the self-renewal of LCSCs, whereas restoration of EpCAM and Sox9 expression in JMJD2D-knockdown LCSCs rescued the tumorsphere formation ability and tumorigenesis, suggesting that JMJD2D enhances LCSC self-renewal through upregulating EpCAM and Sox9 expression. Mechanistically, JMJD2D cooperates with β-catenin/TCF4 and NICD1 to enhance the transcription of EpCAM and Sox9, respectively.

Nowadays, targeting CSC therapy is thought to have great potential for the clinical treatment of cancer patients (37). Many signaling pathways had been reported to be involved in regulating CSC self-renewal, including Wnt/β-catenin, TGF-β, Notch, Hedgehog, NF-κB, and IL6/STAT3 pathways. Among them, Wnt/β-catenin, Notch，and Hedgehog are the main signaling pathways that regulate the maintenance and survival of CSCs and attract much attention. Conventional targeting therapeutics against LCSCs usually target one signaling pathway, which may induce the compensatory activation of other CSC-related signaling pathways and lead to the chemoresistance of tumors. Therefore, it is ideal to find a therapeutic target that can regulate multiple CSC-related signaling pathways simultaneously. In this study, we found that JMJD2D could promote LCSC self-renewal through Wnt and Notch signaling pathways. Moreover, we recently reported that JMJD2D could severe as a coactivator to enhance Hedgehog signaling to promote colorectal cancer progression (25). Hedgehog signaling pathway is also very important for CSC self-renewal (14). We found that knockdown of JMJD2D in HepG2 could inhibit Hedgehog signaling pathway as demonstrated by Gli2 downregulation (Fig. S7). Taken together, JMJD2D may promote CSC self-renewal through activating Wnt, Notch, and Hedgehog signaling pathways simultaneously. Therefore, targeting JMJD2D could attenuate CSC self-renewal through simultaneously inhibiting Wnt, Notch, and Hedgehog signaling pathways, which have great potential for the clinical treatment of liver cancer patients.

As a demethylase, JMJD2D reduced H3K9me3 levels at the promoter region to increase the recruitment of TCF4/β-catenin and NICD1 to the EpCAM and Sox9 promoters and to facilitate the transcription of EpCAM and Sox9, respectively. Therefore, JMJD2D can be targeted by a demethylase inhibitor to suppress the expression of EpCAM, Sox9, c-Myc, Hes1, and Gli2. Indeed, treatment of a JMJD2D inhibitor 5-c-8HQ suppressed the expression of EpCAM, Sox9, c-Myc, Hes1, and Gli2, as well as LCSC tumorsphere formation ability and LCSC orthotopic tumor growth *in vivo*, indicating that 5-c-8HQ treatment can simultaneously inhibit Wnt, Notch, and Hedgehog signaling pathways to inhibit the self-renewal and tumorigenesis of LCSCs. Collectively, our study shows the proof-of-concept use of the JMJD2D inhibitor to target LCSCs for liver cancer therapy.

## Experimental procedures

### Plasmids

Human EpCAM promoter (–2105/+190) reporter and human Sox9 promoter (–1992/+387) reporter were constructed in the pGL3-basic vector for the promoter–reporter assays. The expression plasmids of β-catenin, TCF4, NICD1, and JMJD2D were constructed using HepG2 cDNA as original templates in the pCMV5, pGEX-5X-1, and pLV vectors.

### Antibodies and drugs

The antibody used for western blot analysis are listed as follows: JMJD2D antibody (Abcam, Cat# ab93694, 1:1000); β-actin antibody (Sigma Aldrich, Cat# A5441, 1:1000); EpCAM antibody (Cell Signaling Technology, Cat# 93790, 1:1000); β-catenin (Cell Signaling Technology, Cat# 8480, 1:1000); TCF4 (Cell Signaling Technology, Cat# 2569, 1:1000); Flag-tag antibody (Sigma, Cat# F1804, 1:1000); Myc-tag antibody (ABclonal, Cat# AE070, 1:1000); H3K9me3 antibody (Abcam, Cat# ab8898, 1:1000); Sox9 (Abcam, Cat# ab182579, 1:1000); Notch1 (Cell Signaling Technology, Cat# 3608, 1:1000); HA-tag antibody (Thermo Fisher Scientific, Cat# 26183, 1:1000). The JMJD2D inhibitor 5-c-8HQ (HY-12304) was purchased from MCE. Lipofectamine 2000 (Cat #11668019) was purchased from Invitrogen.

### Cell culture

Human liver cancer cell lines HepG2, Huh-7, mouse liver cancer cell line Hepa1-6, and HEK293 T were cultured in high-glucose DMEM (HyClone) supplemented with 10% fetal bovine serum (FBS) and penicillin-streptomycin. All cell lines were maintained in a humidified chamber with 5% CO_2_ at 37 °C.

### RNA interference

Specific (siJMJD2D-1: 5′-AUCCAAAUUGCAGCAUAAUTT-3′ and siJMJD2D-2: 5′-CCAGGCAGUCUUAUGACAATT-3′) and nonspecific (5′-UUCUCCGAACGUGUCACGUTT-3′) siRNAs were purchased from GenePharma. siRNAs were transfected by Lipofectamine 2000 (Invitrogen) according to the manufacturer’s instructions.

### Knockdown and knockout of JMJD2D

Two different human JMJD2D shRNA plasmids (sh2D-1 and sh2D-2) were generated by inserting two different human JMJD2D-specific targeting sequences 5′-CAGATTATCCACCCGTCAAAT-3′ and 5′-GGTTAGCGTAACCTGGTATAT-3′ into pLKO vector plasmids. sgRNA targeting Mouse JMJD2D CDS was generated by inserting sequences 5′-TGCTTTGGTCACCCGCCGGG-3′ to Lenti-V2 vector. Establishment of stable knockdown or knockout cell lines was described previously (38).

### Cell proliferation assay

MTT assays were used to measure cell proliferative rate as described previously (38).

### Tumorsphere culture and serial passage assay

Tumorsphere culture was performed as described previously (39). For primary tumorsphere culture, cells were seeded at 3000 cells per well. After 5–7 days, primary tumorspheres were centrifuged and dissociated with 0.05 % trypsin-EDTA (Invitrogen) and then sieved (40 μm) to obtain single cell. Single cells derived from primary tumorspheres were seeded at 3000 cells per well for secondary tumorspheres, then single cells derived from secondary tumorspheres were seeded at 3000 cells per well for tertiary tumorspheres.

### *In vitro* limiting dilution assay

*In vitro* limiting dilution assay was performed as described previously (40).

### Reverse transcription and real-time PCR

Total RNA was isolated using trizol reagent (Invitrogen) according to the manufacturer’s instructions. cDNA was obtained from 2 μg of total RNA using a reverse transcription kit (Toyobo). Real-time PCR was performed using universal SYBR Green Master (Roche Applied Science), and relative quantification was achieved by normalization to β-actin. Sequences of the primers used for real-time PCR are listed on Table S1.

### Luciferase reporter assay

The EpCAM or Sox9 promoter reporter was transfected into cells, the renilla reniformis luciferase reporter serviced as an internal control. The firefly luciferase activity of individual sample was standardized relative to the renilla luciferase activity and expressed in units (-fold change) relative to control sample value assigned a unit of 1. The activities of EpCAM and Sox9 promoter reporters were analyzed by the luciferase reporter assay system (Promega, Madison, US).

### Coimmunoprecipitation (Co-IP) and GST pull-down assay

The interactions of JMJD2D with β-catenin, TCF4, and NICD1 in HepG2, Huh-7, and Hepa1-6 were analyzed by the Co-IP assay and GST pull-down assay as described previously (38).

### Chromatin immunoprecipitation (ChIP) assay

ChIP assay was performed as previously described (41). Briefly, control and JMJD2D-knockdown cells were fixed with 1% formalin for 10 min, and then chomatin was immunoprecipitated using antibody for JMJD2D (Abcam, Cat# ab93694, 1:50), TCF4 (Cell Signaling Technology, Cat# 2569, 1:50), β-catenin (Cell Signaling Technology, Cat# 8480, 1:50), NICD1 (Cell Signaling Technology, Cat# 3608, 1:50), and H3K9me3 (Abcam, Cat# ab ab8898, 1:50). The ChIP DNA was isolated and detected by quantitative real-time PCR using specific EpCAM promoter primer (TCF4-binding element 1 (TBE1): 5′-TACATTAATCAACTTGCCGG-3′ and 5′-CAGAGCAAGACTCCATCTC-3′; TCF4-binding element 2 (TBE2): 5′-TCAACTTGCCGGCACTTCA-3′ and 5′-AGCAATTTTAAGATTCAGC-3′; and Sox9 promoter primer (5′-TAAGTCGGGAAGGTTCCTTG-3′ and 5′-GGCTTGCAGAATGCGGAACA-3′.

### Tumor graft

Four to six-week-old male nude mice and C57BL/6 mice were obtained from the Laboratory Animal Center of Xiamen University. JMJD2D-knockdown/knockout cells or control cells derived from tumorspheres were subcutaneously injected into the dorsal flanks of mice. The tumor size was measured along two perpendicular axes every 2 days from day 8 after cell injection using a vernier caliper. The volume of the tumor was calculated using the formula: Volume = Length × Width^2^ × 0.52. All experimental procedures involving animals were performed in accordance with animal protocols approved by the Laboratory Animal Center of Xiamen University.

### *In vivo* limiting dilution assay

For *in vivo* limiting dilution assay, four to six-week-old male BALB/c nude mice or C57BL/6 mice were injected with 1 × 10^2^, 5 × 10^2^, 1 × 10^3^, and 1 × 10^4^ cells derived from tumorspheres into the dorsal flanks of mice. After 2 months, the number of tumors was counted.

### Detection of circulating liver cancer stem-like cells

Detection of circulating LCSCs was performed as previously described (42). Briefly, GFP-labeled LCSCs (1 × 10^6^) dissociated from spheroids were suspend in PBS and subsequently injected into the lateral tail veins of mice in a volume of 0.2 ml. Whole blood specimens were collected *via* heart acupuncture at 36 h after transplantation. Erythrocytes were lysed in RBC Lysis Buffer and peripheral blood mononuclear cells (PBMCs contain circulating tumor cells) were enriched from the blood. Circulating LCSCs were analyzed by detecting GFP-positive cells using flow cytometry on a BD LSRFortessa (BD Biosciences, San Jose, CA).

### LCSC GFP-labeling process

Lentiviruses carrying GFP sequence were packed through transfecting lentiviral transfer vectors pLV-GFP and three additional plasmids (pMDL, pREV and pVSVG) into 293T cells. Cancer cells were infected with lentiviruses carrying GFP sequence for 72 h, and then cancer cells were seeded in CSC culture media for 7 days to form tumorsphere. Tumorsphere was disassociated into single cell suspensions by trypsin, and then fluorescence-activated cell sorter (FACS) was used for isolation and enrichment of GFP-labeled (GFP-positive) LCSCs. Almost all of these cells were GFP-positive under the fluorescence microscope (data not shown).

### Orthotopic graft tumor model

The orthotopic graft tumor model was performed as previously describe (43). Briefly, LCSCs (1 × 10^6^), disassociated from cultured HepG2 and Hepa1-6 spheroids and suspended in 30 μl PBS, were slowly injected into the left lobe of the liver using a 28-gauge needle. After cell injection, a small piece of sterile gauze was placed on the injection site, and light pressure was applied for 5 min to prevent bleeding. The abdomen was then closed with a 6-0 silk suture. After surgery, animals were kept in a warm cage, observed for 1–2 h and subsequently returned to the animal room after full recovery from the anesthesia. After 3 days of inoculation of cancer cells, mice were randomly divided into three groups and then gavaged daily for 12 days with 1% sodium carboxymethyl cellulose (Control), 10 mg/kg 5-c-8HQ (low-dose group), and 20 mg/kg 5-c-8HQ (high-dose group) dissolved in 1% sodium carboxymethyl cellulose, respectively. After 12 days of drug administration, mice were sacrificed, and then tumors and organ tissues were collected for section and storage in a –80 °C refrigerator for subsequent analysis.

### Immunohistochemistry

Immunohistichemistry analysis was performed as previously described (41). The primary antibody used for immunohistochemistry analysis was Ki67 (ab15580; Abcam).

### Western blot analysis

Western blot analysis was performed as previously described (41). The antibodies used for western blot analysis are listed in “Antibodies and drugs.”

### Statistical analysis

All data are shown as the mean ± SD (Standard deviation) of at least three replicates. The statistical analysis of the difference between two groups was determined using two-tailed Student’s *t*-tests, and multiple group comparisons were performed using one-way or two-way ANOVA followed by Tukey’s post hoc test. For all figures, ns (*p* > 0.05); ∗*p* < 0.05; ∗∗*p* < 0.01; ∗∗∗*p* < 0.001; ∗∗∗∗*p* < 0.0001.

## Data availability

All data described are contained within the article and the supporting information file. This article contains Table S1 and Figs. S1–S9.

## Conflict of interest

The authors declare that they have no conflicts of interest with the contents of this article.
