# Supplementary material for: Histone demethylase JMJD2D promotes the self-renewal of liver cancer stem-like cells by enhancing EpCAM and Sox9 expression
Source: J Biol Chem. 2020 Dec 3;296:100121. doi: 10.1074/jbc.RA120.015335 (PMC7948496; doi:10.1074/jbc.RA120.015335)
Supplement: Table S1 and Figs. S1–S9 [file mmc1.pdf]

Supporting Information

Histone demethylase JMJD2D promotes the self-renewal of liver cancer stem-like cells by enhancing EpCAM and Sox9 expression

Yuan Deng[1, #], Ming Li[2, #], Minghui Zhuo[1], Peng Guo[1], Qiang Chen[1], Pingli Mo[1], Wengang Li[2,*], Chundong Yu[1,*]

[1]State Key Laboratory of Cellular Stress Biology, Innovation Center for Cell Biology, School of Life Sciences, Xiamen University, Xiamen, China;
[2]Department of Hepatobiliary Surgery, Xiang'an Hospital of Xiamen University, School of Medicine, Xiamen University, China;

[#]These authors contributed equally to this work
*Correspondence: Chundong Yu, State Key Laboratory of Cellular Stress Biology, Innovation Center for Cell Biology, School of Life Sciences, Xiamen University, Xiamen, Fujian, China 361005. Tel: 86-592-2182013, Fax: 86-592-2182083, E-mail: cdyu@xmu.edu.cn; Wengang Li, Xiamen City Key Laboratory of Biliary Tract Diseases, Xiang'an Hospital of Xiamen University, Xiamen, China. Email: lwg11861@163.com

Running Title: JMJD2D promotes the self-renewal of LCSCs

Keywords: JMJD2D; Wnt signaling; Notch signaling; H3K9me3; Liver cancer stem-like cell; 5-c-8HQ

Figure S1

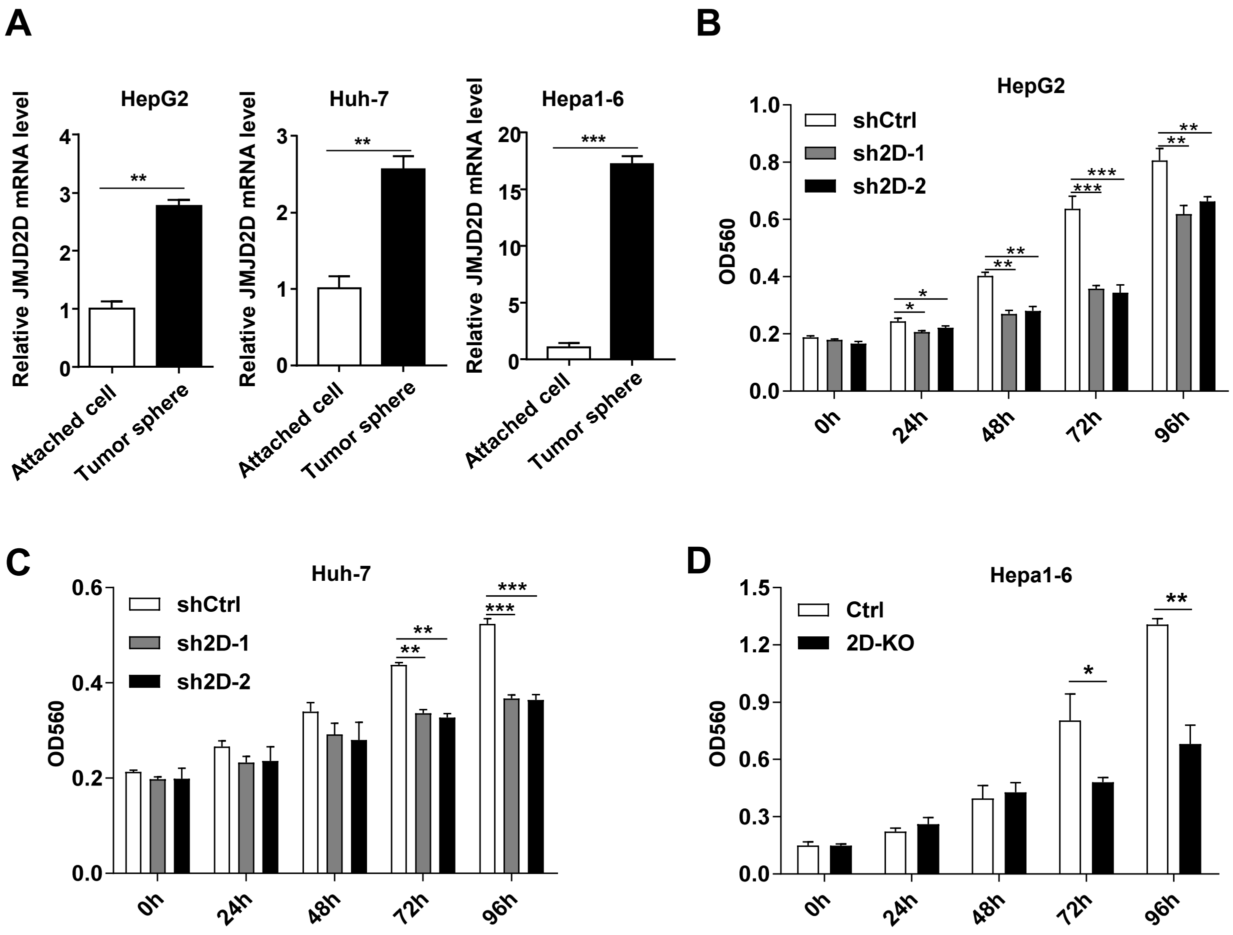

Figure S1. JMJD2D mRNA is upregulated in LCSCs and knockdown or knockout of JMJD2D reduces liver cancer cell proliferation. *A*, JMJD2D mRNA were upregulated in LCSCs. LCSCs were enriched by inducing hepatoma spheroid formation and then real-time PCR was performed to detect the mRNA expression of JMJD2D in attached liver cancer cells and tumor spheres. *B* and *C*, knockdown of JMJD2D reduced the proliferation of HepG2 and Huh-7 cells. *D*, knockout of JMJD2D reduced the proliferation of Hepa1-6 cells. These experiments were performed at least three times with similar results. *, $p<0.05$; **, $p<0.01$; ***, $p<0.001$.

Figure S2

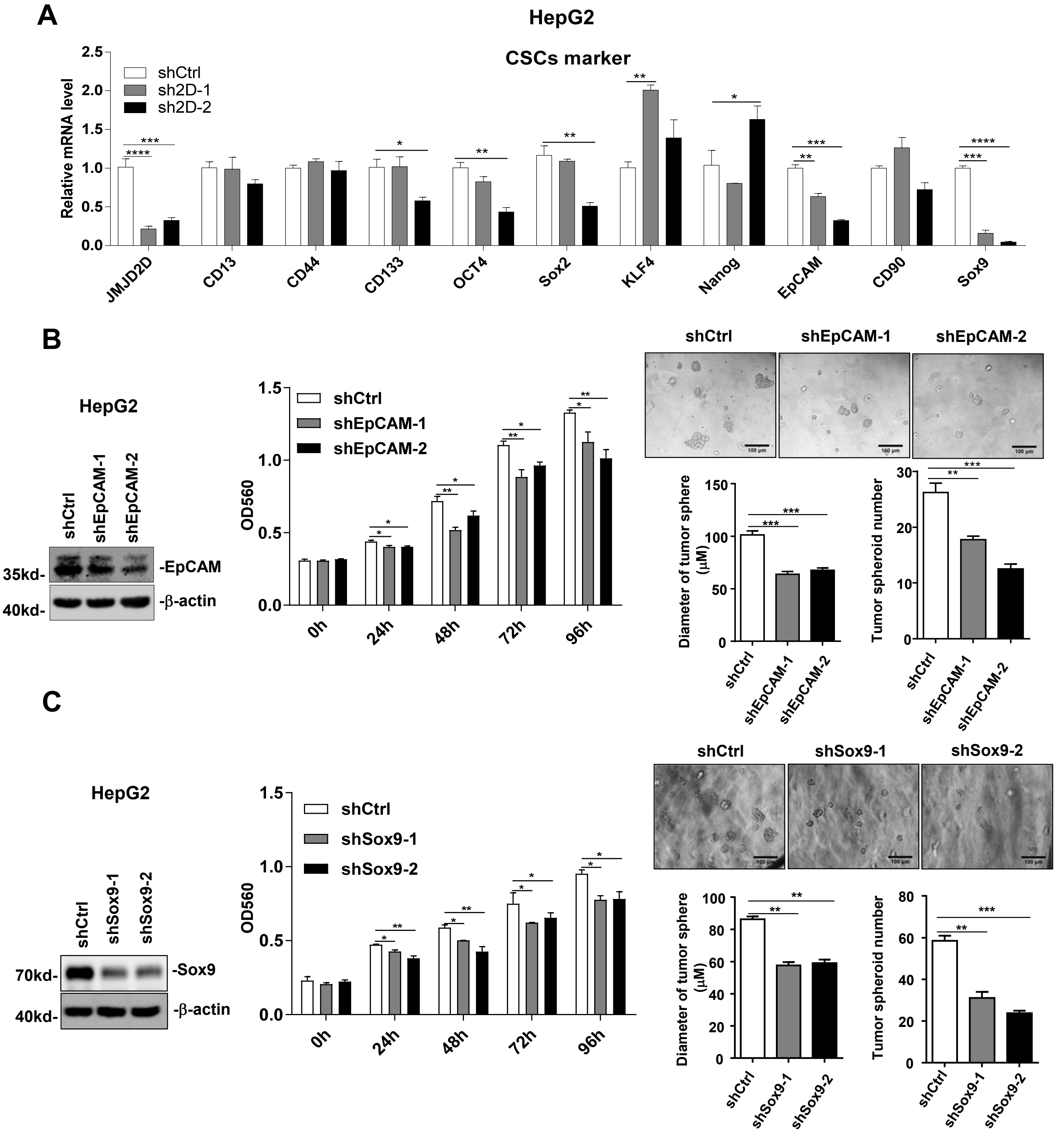

Figure S2. Downregulation of EpCAM and Sox9 is responsible for the inhibitory effects of JMJD2D knockdown on the self-renewal of LCSCs. *A*, the mRNA levels of EpCAM and Sox9, but no other CSC markers, were significantly reduced in two JMJD2D-knockdown HepG2 cell lines. *B*, knockdown of EpCAM inhibited the proliferation and tumorsphere formation ability of HepG2. *C*, knockdown of Sox9 inhibited the proliferation and tumorsphere formation ability of HepG2. These experiments were performed at least twice with similar results. *, $p<0.05$; **, $p<0.01$; ***, $p<0.001$; ****, $p<0.0001$.

Figure S3

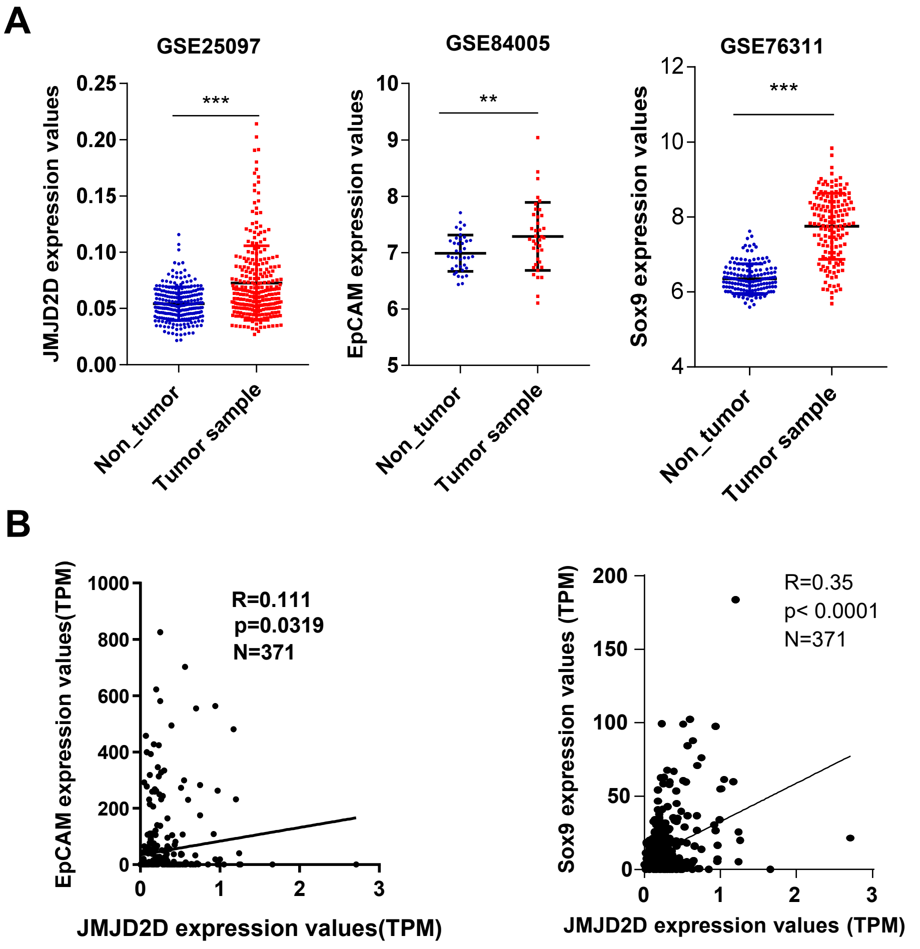

Figure S3. The expression of JMJD2D, EpCAM, and Sox9 is upregulated in human liver cancer specimens with positive correlation. *A*, GEO database showed that JMJD2D, EpCAM and Sox9 were upregulated in liver cancer tissues compared with adjacent non-tumor tissues. *B*, TCGA database showed that the mRNA levels of JMJD2D were positively correlated with EpCAM and Sox9. **, $p<0.01$; ***, $p<0.001$.

Figure S4

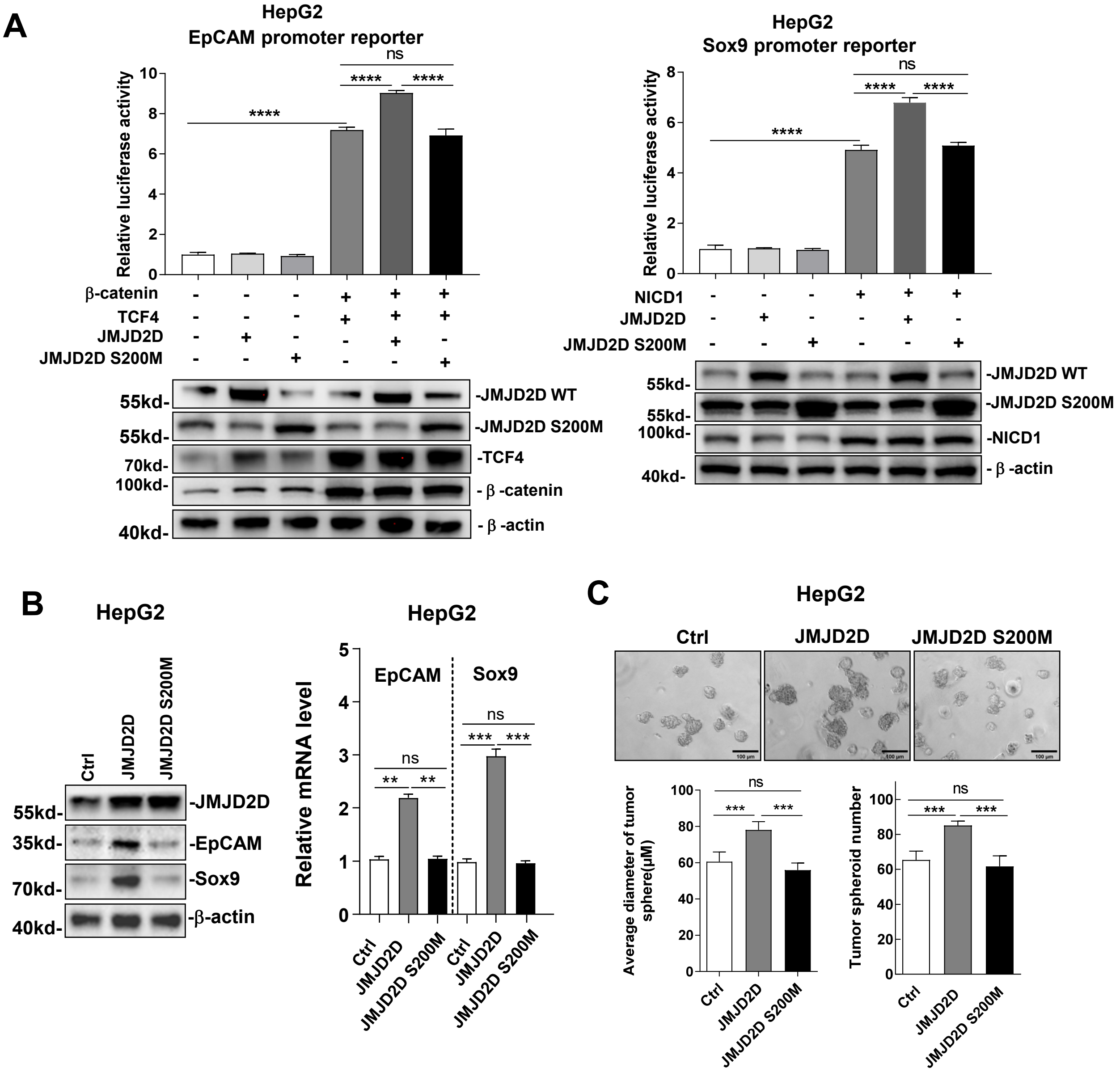

Figure S4. The demethylase activity of JMJD2D is required for the transcription of EpCAM and Sox9 to promote the self-renewal of LCSCs. *A*, JMJD2DS^{200M} mutant failed to cooperate with β-catenin/TCF4 and NICD1 to enhance the promoter activities of EpCAM, Sox9. *B*, JMJD2DS^{200M} mutant failed to increase the protein and mRNA levels of EpCAM and Sox9. *C*, JMJD2DS^{200M} mutant failed to enhance tumorsphere formation ability. These experiments were performed at least twice with similar results. **, $p<0.01$; ***, $p<0.001$; ****, $p<0.001$.

Figure S5

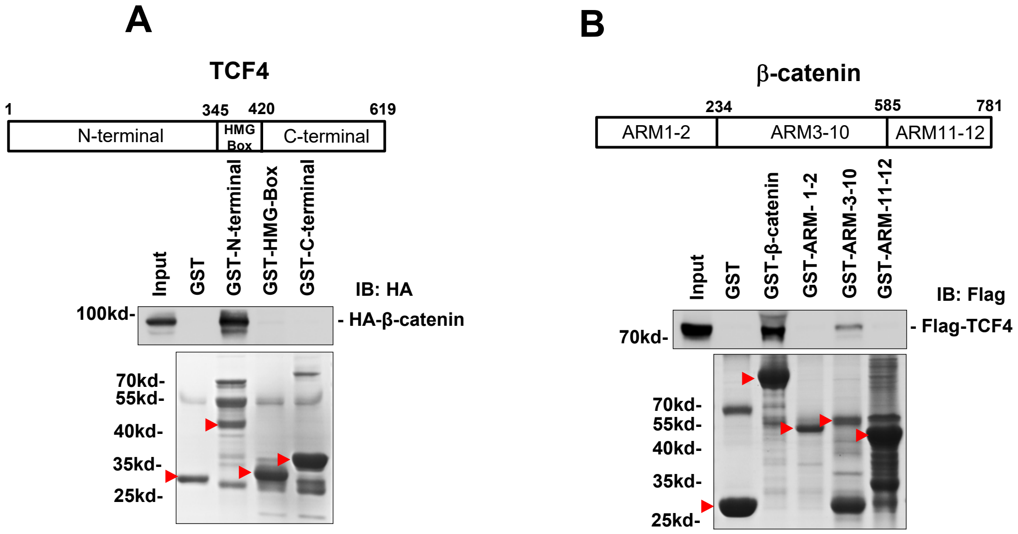

Figure S5. TCF4 interacts with β-catenin. *A,* GST pull-down analysis of the interaction between β-catenin and different domains of TCF4. *B*, GST pull-down analysis of the interaction between TCF4 and the different domains of β-catenin. These experiments were performed at least three times with similar results.

Figure S6

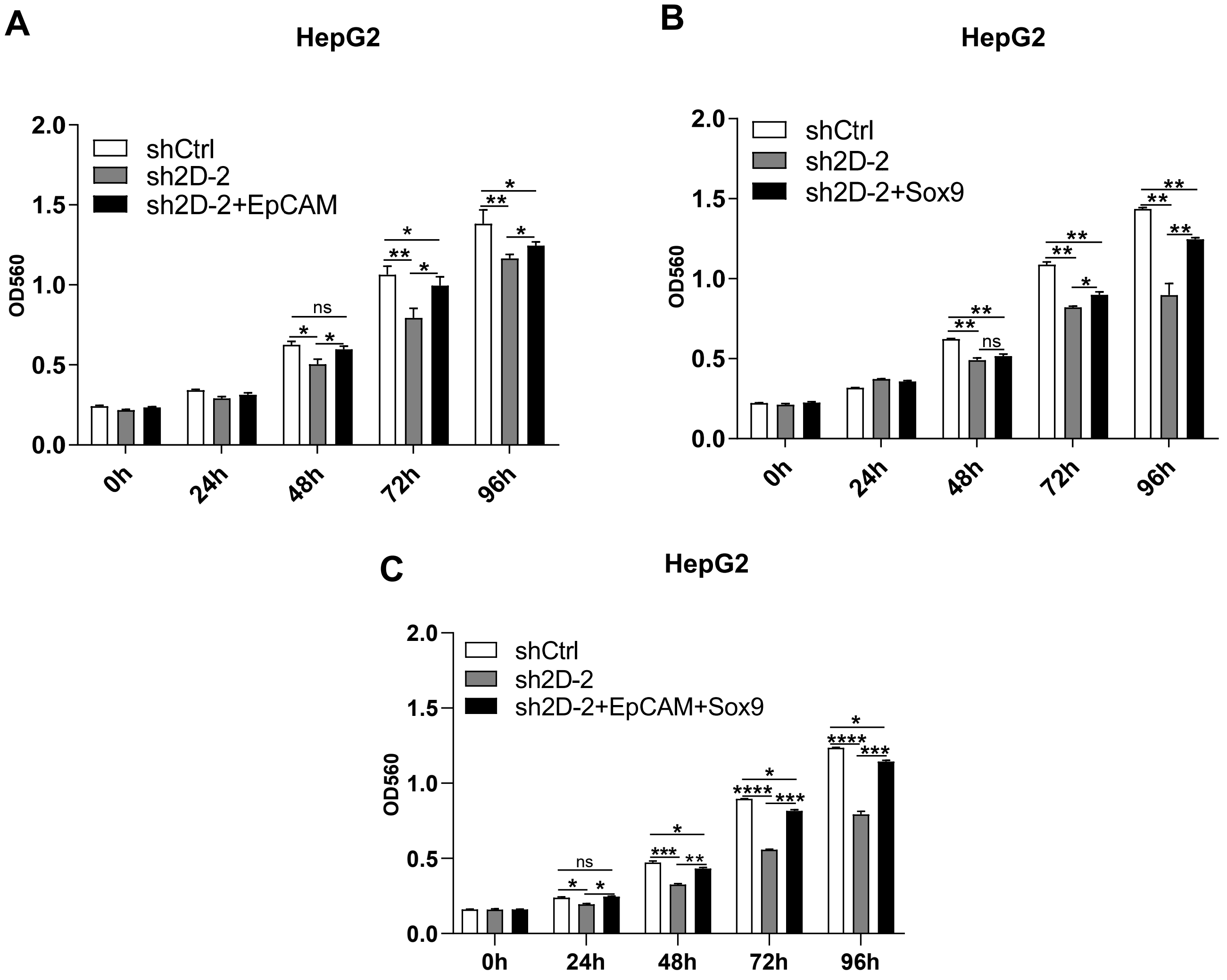

Figure S6. Ectopic expression of EpCAM, Sox9, and EpCAM plus Sox9 partially rescued the proliferation of JMJD2D-knockdown cells under the adherent culture conditions, respectively. *A*, ectopic expression of EpCAM partially rescued the proliferation of JMJD2D-knockdown cells. *B*, ectopic expression of Sox9 partially rescued the proliferation of JMJD2D-knockdown cells. *C*, simultaneous restoration of EpCAM and Sox9 expression rescued the proliferation of JMJD2D-knockdown cells more efficiently. These experiments were performed at least twice with similar results. *, $p<0.05$; **, $p<0.01$; ***, $p<0.001$; ****, $p<0.0001$.

Figure S7

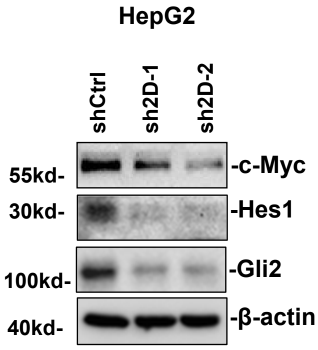

Figure S7. Knockdown of JMJD2D downregulates the protein expression of c-Myc, Hes1, and Gli2 in HepG2 cells. These experiments were performed at least three times with similar results.

Figure S8

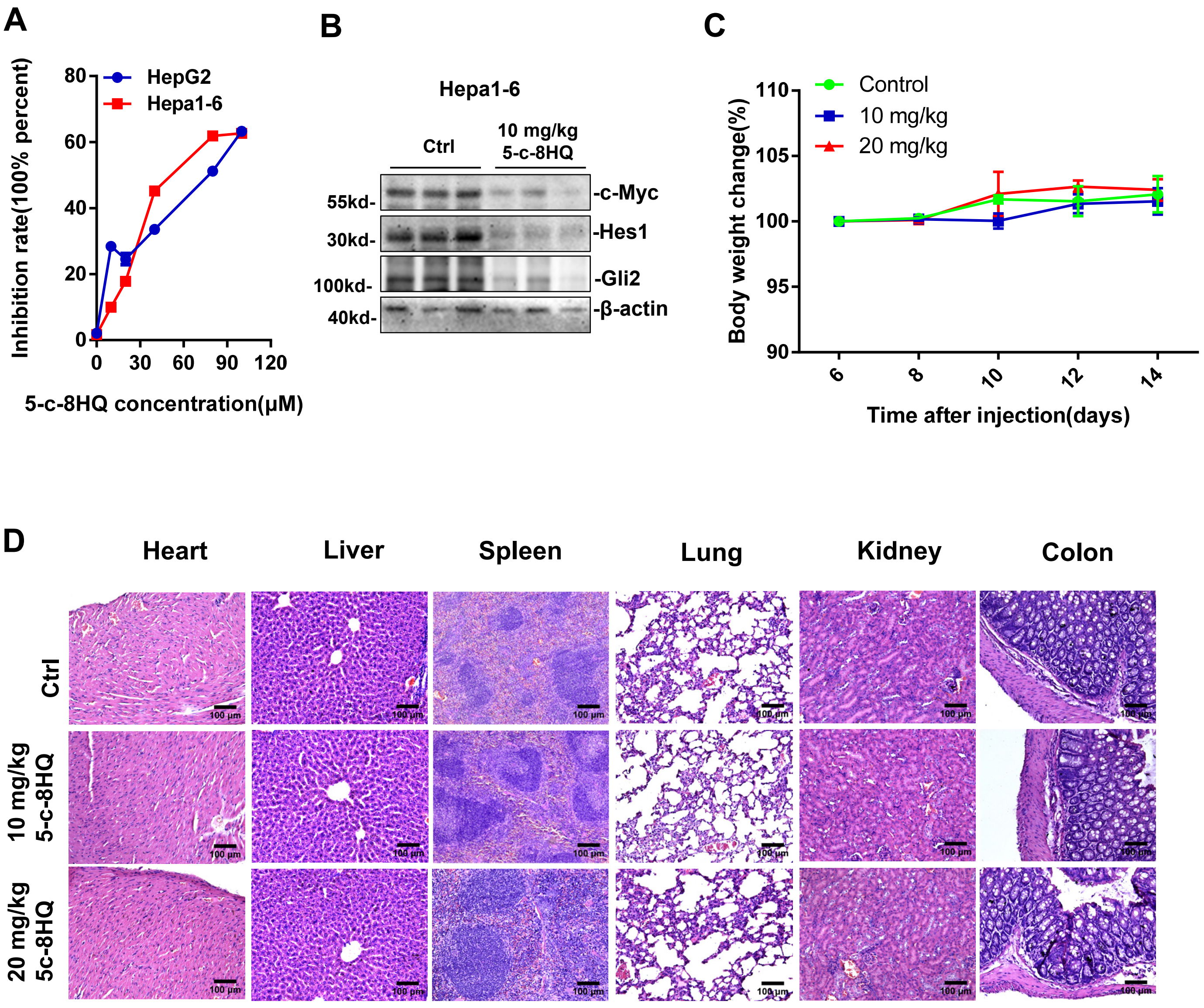

Figure S8. The effects of the JMJD2D inhibitor 5-c-8HQ treatment. *A*, 5-c-8HQ inhibited the proliferation of liver cancer cells HepG2 and Hepa1-6. *B*, 5-c-8HQ suppressed the expression of c-Myc, Hes1, and Gli2 in orthotopic tumor. *C*, 5-c-8HQ treatment did not change the mouse body weight in orthotopic graft tumor model. *D*, 5-c-8HQ treatment showed no obvious toxicity to mouse major organs (H&E staining of the heart, liver, spleen, lung, kidney, and colon) in orthotopic graft tumor model . These experiments were performed at least twice with similar results.

Figure S9

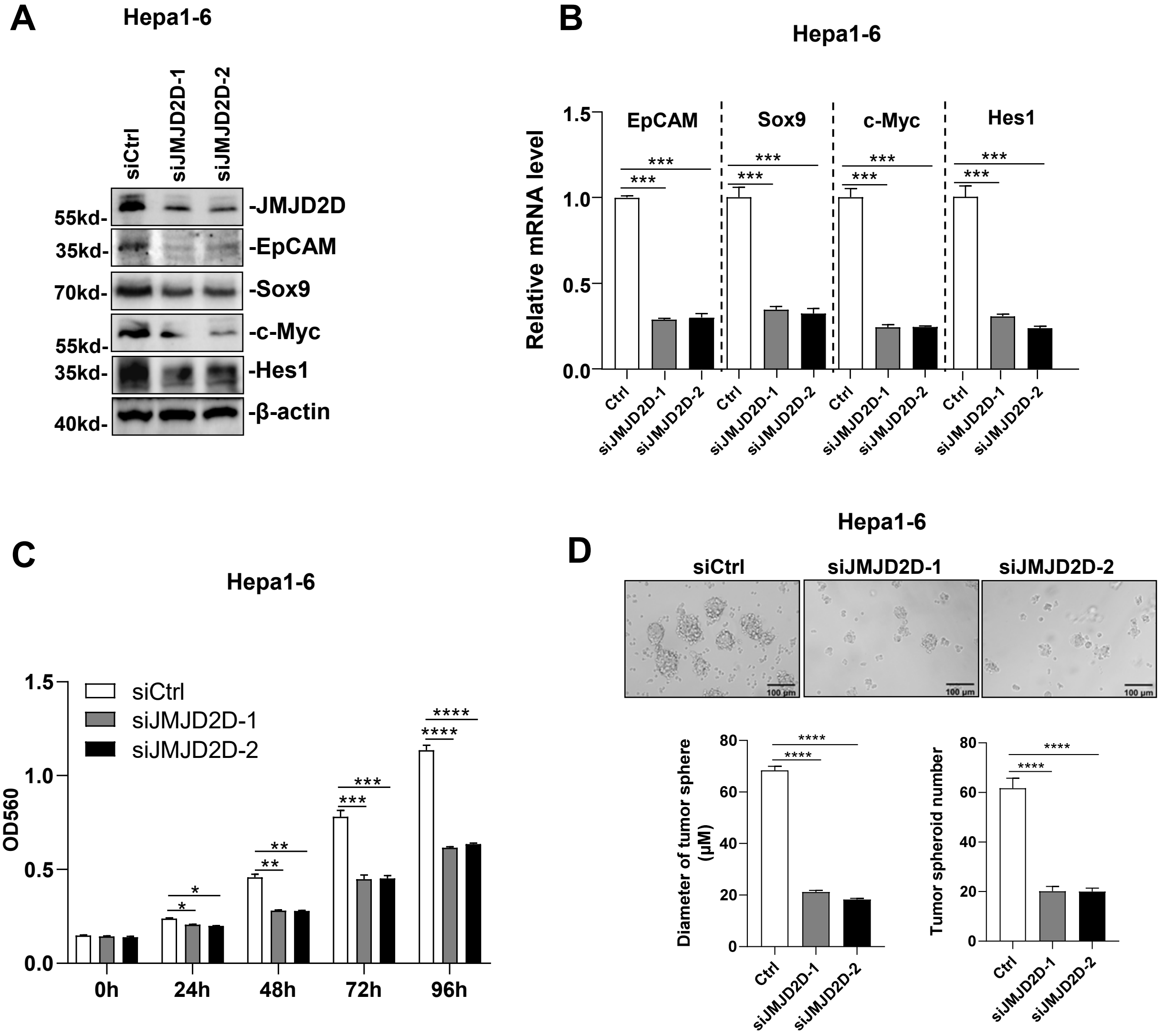

Figure S9. The effects of transient knockdown of JMJD2D by siRNA on Hepa1-6 cells. *A* and *B*, transient knockdown of JMJD2D reduced the protein and mRNA levels of EpCAM, Sox9, c-Myc, and Hes1 in Hepa1-6 cells. *C* and *D*, transient knockdown of JMJD2D inhibited the proliferation and tumorsphere formation of Hepa1-6 cells. These experiments were performed at least twice with similar results. *, $p<0.05$; **, $p<0.01$; ***, $p<0.001$; ****, $p<0.0001$.

Table S1

Table S1 q-PCR primer used in this study (h: human; m: mouse).

q-PCR primer	Forward primer	Reverse primer
h-CD13	TTCAACATCACGCTTATCCA	AGTCGAACTCACTGACAAT
h-JMJD2D	ACGCTATGACCTGTGGAA	CTCCTGGGTAACTGGACTT
m-JMJD2D	GCCTATAGCCACGGTAAGTAAC	GAGTCAGACTTCTGGACATCATC
h-CD44	CGCTTTGCAGGTGTATTCCA	ACCACGTGCCCTTCTATGAA
h-CD133	GCCTCTGGTGGGGTATTTCT	CGACTCCTTTTGATCCGGGT
h-OCT4	CTTGAATCCCGAATGGAAA	CCTTCCCAAATAGAACCCCC
h-Sox2	GCCGAGTGGAAACTTTTGT	GCAGCGTGTACTTATCCTTC
h-Klf4	CCCACATGAAGCGACTTCC	CAGGTCCAGGAGATCGTTG
h-Nanog	TTTGTGGGCCTGAAGAAAA	AGGGCTGTCCTGAATAAGCA
h-EpCAM	ACCTGACAGTAAATGGGGA	CAGCCTTCTCATACTTTGCC
m-EpCAM	CTGGCGTCTAAATGCTTGGC	CCTTGTCGGTTCTTCGGACTC
h-c-Myc	ACCACCAGCAGCGACTCTG	AGCAGAGGTGATCCAAGAC
h-CD90	TCACCCATCCAGTACGAGT	GGAGCGGTATGTGTGCTCAG
h-Sox9	AGCGAACGCACATCAAGAC	CTGTAGGCGATCTGTTGGGG
m-Sox9	AGTACCCGCATCTGCACAAC	ACGAAGGGTCTCTTCTCGCT
h-β-actin	CATGTACGTTGCTATCCAGG	CTCCTTAATGTCACGCACGA
m-β-actin	GTGACGTTGACATCCGTAAAGA	GCCGGACTCATCGTACTCC
